# Social and Poor-Law Problems

**Published:** 1908-05-16

**Authors:** 


### SOCIAL AND POOR-LAW PROBLEMS.

### PHTHISIS, MUNICIPALITIES, AND GUARDIANS.

Until phthisis is a notifiable disease it is difficult to get proper control of it, but as a knowledge of its seriousness, not only to the sufferer but also to his family becomes generally known, there comes a growing readiness to accept, and even to demand help in its treatment. The question then arises, to whom should the sufferer look for the assistance he needs—to voluntary charity, to the municipality, or to the Poor-law? Undeniably the majority of consumptives would prefer the first. There is a graciousness in the voluntary idea that "blesses him that gives and him that takes," and in some subtle way the recipient does not feel humiliated by accepting help from unknown donors if their contributions do not come through the Poor-law. But when voluntary charity is insufficient either the municipality or the guardians must step in. As in either case the money is drawn from the same source, this might seem a distinction without a difference, but again self-respect comes in, and those who have no hésitation in going into a municipal hospital, as rate-payers, object to accepting help from the hands of the guardians as paupers. Especially when phthisis is traceable to one of the standard industries of the place, it is better that help should be given by the municipality—if it is not given as a free-will offering by employers. Thus, in certain parts of the country miners' phthisis is found as a result of work in the coalpits. Stonemason's lung is another trade disease, and, in spite of modern improvements in the works, steel grinders suffer from the work they do. All these forms of disease are not equally susceptible to remedial treatment, but there are few cases in which some benefit would not be derived, and in very few can treatment be given at home, though instruction in hygiene may in time teach those exposed to danger what precautions are necessary. Meanwhile, those affected need treatment. At present many evade it because they can, after a fashion, go on working for a long time—usually until treatment can do nothing for them. If phthisis were notifiable and treatment in a suitable sanatorium made compulsory if the medical officer of health thought it was necessary, patients and their friends would submit to the inevitable, as they do at present with scarlet fever and the like. And the knowledge that if the disease took a firm hold the patient would be removed, might make those threatened with it more careful to avoid the onset.

